# Effects of combining Taxol and cyclooxygenase inhibitors on the angiogenesis and apoptosis in human ovarian cancer xenografts

**DOI:** 10.3892/ol.2012.1086

**Published:** 2012-12-19

**Authors:** WEI LI, YUN-XIAN TANG, LIANG WAN, JIA-HUI CAI, JUN ZHANG

**Affiliations:** Department of Gynecology, Nanjing Medical University of Hangzhou Hospital, Hangzhou, Zhejiang 310006, P.R. China

**Keywords:** ovarian cancer, SC-560, celecoxib, Taxol, vascular endothelial growth factor, microvessel density, cell apoptosis

## Abstract

The present study aimed to investigate the combined effects of Taxol and cyclooxygenase (COX) inhibitors on angiogenesis and cell apoptosis of SKOV-3 human ovarian carcinoma cell xenograft-bearing mice. The experiments were continued for 28 days. Animals were treated with 3 mg/kg SC-560 (a COX-1-selective inhibitor) alone, 100 mg/kg celecoxib (a COX-2-selective inhibitor) alone or SC-560/celecoxib by gavage twice a day, 20 mg/kg Taxol alone intraperitoneally once a week or in combination with SC-560 or celecoxib or SC-560/celecoxib/Taxol for three weeks. The mRNA levels of vascular endothelial growth factor (VEGF) was determined by reverse transcription-polymerase chain reaction (RT-PCR). The microvessel density (MVD) of ovarian carcinoma was determined by immunohistochemistry with anti-CD_34_ as the label. The apoptotic index was detected by the terminal deoxynucleotidyl transferase-mediated deoxyuridine triphosphate nick end labeling (TUNEL) method. The MVD value and apoptotic index in the SC-560/Taxol group were notably inhibited compared with the Taxol group (P<0.001). Moreover, the VEGF mRNA levels, MVD value and apoptotic index in the SC-560/Taxol group were significantly different from the celecoxib/Taxol group (P<0.05, P<0.05 and P<0.001, respectively). The present study demonstrated that SC-560 enhances the anti-angiogenic and pro-apoptotic effects of Taxol and these effects are better than with celecoxib.

## Introduction

Ovarian cancer is associated with a high mortality due to the absence of effective screening strategies to identify patients at high risk or who have already developed early neoplastic lesions still amenable to treatment (1). The current management of advanced ovarian cancer includes cytoreductive surgery followed by combination chemotherapy; however, the long-term survival of ovarian cancer patients remains unsatisfactory (2). Despite advances in surgery and chemotherapy, novel treatment strategies are required to further benefit patients (3). Taxanes are widely used to treat patients with cancer of the lung, breast, stomach, endometrium or ovary (4). At present, chemotherapy in combination with Taxol is the standard first-line therapy for patients with advanced ovarian cancer (5); however, tolerance to Taxol in ovarian cancer cells has been observed (6) and the mechanisms of resistance are not yet fully understood.

Subbaramaiah *et al* observed that taxanes have the ability to promote transcription of the cyclooxygenase (COX)-2 gene and to stabilize the COX-2 messenger RNA transcript (7). Sorokin (8) identified that enforced expression of COX-2 causes enhancement in multidrug resistance (MDR) expression and functional activity. Therefore, upregulation of COX-2 induced by taxanes may attenuate the antitumor effect of taxanes. COX-2 is one of the key enzymes that catalyze the rate-limiting step in prostaglandin (PG) biosynthesis from arachidonic acid, and an elevated expression of COX-2 is associated with tumor growth, invasion (9), migration (10), increased stage, reduced survival rate (11) and chemoresistance (12) of ovarian cancers. A number of studies have demonstrated that COX-2-selective inhibitors inhibit the COX enzymes, downregulate the level of PGE_2_ and decrease the production of vascular endothelial growth factor (VEGF) in tumors. Additionally, they have anti-angiogenic effects on the neovasculature and attenuate tumor growth (9,13). Therefore, early results revealed enhanced anticancer activity from the addition of COX-2 inhibitors to taxane in non-small cell lung cancer (NSCLC) and human endothelial cells by inhibiting PG production and enhancing anti-angiogenic effects (14,15).

COX-1, another key enzyme that catalyzes the rate-limiting step in PG biosynthesis from arachidonic acid, is overexpressed in ovarian cancer (16) and is considered the dominant pathway responsible for generating PGs in epithelial ovarian cancers (17). COX-1-selective inhibitors demonstrate potent antitumor activity in ovarian tumors by influencing cell proliferation and apoptosis and decreasing the production of VEGF in tumors (17,18). In our previous study, we observed that a combination of COX-1 and COX-2-selective inhibitors have better chemopreventive properties on ovarian cancer than when administered alone (19). However, no studies have reported on the addition of COX-1 inhibitors to taxane on ovarian cancer treatment. Consequently, we investigated the effect of combining Taxol and COX inhibitors on tumor growth, angiogenesis and apoptosis in a human ovarian cancer xenograft.

## Materials and methods

### Human ovarian tumors in nude mice

SKOV-3 cells were used for tumor growth studies *in vivo*. The SKOV-3 cells were purchased from the China Center for Type Culture Collection and grown in the recommended media under standard conditions. SKOV-3 cells were implanted subcutaneously in the dorsal skin (2×10^6^ cells) of female athymic nude mice (nu/nu, 7–8 weeks old). When the tumors became visible (7 days after inoculation), the mice were randomly separated into eight groups (n=6): control, SC-560, celecoxib, Taxol, SC-560/Taxol, celecoxib/Taxol, SC-560/celecoxib and SC-560/celecoxib/Taxol. The study was approved by the ethics committee of Nanjing Medical University of Hangzhou Hospital, Hangzhou, China.

### Dose and administration time of drugs

COX inhibitors, SC-560 (Sigma, St. Louis, MO, USA) and celecoxib (Pfizer, Groton, CT, USA) were administered by gavage and Taxol (Bristol-Myers Squibb SRL, Italy) was administered by intraperitoneal injection in a 0.5 ml suspension of 0.5% methylcellulose (Sigma) and 0.025% Tween-20 (Sigma) at a dose of 3 mg/kg (SC-560) and 100 mg/kg (celecoxib) twice a day and 20 mg/kg (Taxol) once a week. The doses of COX inhibitors were selected for their specificity in inhibiting COX isotypes (20). In the control group, mice were treated with physiological saline under similar conditions. Drugs or the vehicle control were administered for a period of 21 days, beginning one week after the tumors became palpable.

### Measurement of tumor volume

The tumor dimensions were measured twice a week using a linear caliper and tumor volume was calculated using the following equation: volume (mm^3^) = a × b^2^/2, where a is the largest diameter and b is the smallest diameter (21). The animals were weighed weekly throughout the study. On day 28, all mice were sacrificed and tumor tissue samples were collected and fixed in 10% phosphate-buffered formalin solution for immunohistology or stored at −80°C until analyzed. The tumor tissue samples were snap-frozen in liquid nitrogen prior to their storage at −80°C.

### Reverse transcription-polymerase chain reaction (RT-PCR) for VEGF mRNA

Total RNA was extracted using TRIzol reagents (Invitrogen Life Technologies, Carlsbad, CA, USA), according to the manufacturer’s instructions. Isolated RNA was electrophoresed through 1.0% agarose-formaldehyde gels to verify the quality of the RNA. The first strand cDNA was generated by reverse transcription. After a sufficient amount of cDNA was obtained, we performed PCR amplification using a real-time PCR cycler (ABI 7500, Applied Biosystems Company, Foster City, CA, USA). VEGF 189, 165 and 121 were routinely detected in this series of ovarian cancer. The sequences of PCR primers were: VEGF 121, 5′-ACTCGGAT GCCGACACGGGA-3′ and 5′-CCTGGCCTTGCTTGCTC CCC-3′; VEGF 165, 5′-CCAGGATCCTCTGCCCGCCT-3′ and 5′-GCGGCTTCCGGCACCTACAG-3′; VEGF 189, 5′-GGCAAAAGTTGCGAGCCGCC-3′ and 5′-TGGATG GACCGGGAGCAGGG-3′; β-actin, 5′-GGGTGACGAGGC CCAGAGCA-3′ and 5′-GGG GCCACACGCAGCTCATT-3′. The amplification system included 50 *μ*l of SYBR-Green mix (32.5 *μ*l), ddH_2_O (14.5 *μ*l), cDNA (2 *μ*l), forward primer (0.5 *μ*l) and reverse primer (0.5 *μ*l). The reaction conditions were as follows: stage 1, 50°C for 2 min (1 cycle); stage 2, 95°C for 5 min (1 cycle); stage 3, 95°C for 0.25 min followed by 60°C for 0.75 min (40 cycles); stage 4, 95°C for 0.25 min, then 60°C for 1 min and lastly, 95°C for 0.25 min followed by 60°C for 0.25 min (1 cycle). The results of real-time PCR were analyzed by the DCt method: ΔCT = CT_selected gene_ − CT_β-actin_. Relative quantitation (RQ) = 2^−ΔCT^ × 100%. The results of real-time PCR were presented as the ratio between the selected genes and β-actin transcripts.

### Immunohistochemistry for MVD

Formalin-fixed paraffin-embedded tumor sections (6 *μ*m) were subjected to immunostaining using CD_34_ antibodies (Santa Cruz Biotechnology Inc., Santa Cruz, CA, USA). Sections were deparaffinized and hydrated by sequential immersion in xylene and grade alcohol solutions. The sections were then incubated with 3% hydrogen peroxide in methanol solution for 34 min to block endogenous peroxidase activity. For antigen retrieval, slides were pressured in the pressure cooker for 2×10 min. For CD_34_ staining, the sections were immersed in normal goat serum for 34 min. Immunohistochemical staining was performed using the streptavidin-biotin method. Microvessel density (MVD) was evaluated according to the method first described by Weidner *et al*(22). The entire tumor section was first carefully scanned at low magnification with light microscopy (magnification, ×40) to find the area that presented the most intense neovascularization. Since the immunohistochemistry of CD_34_ demonstrated slight heterogeneity within the same tumor, the five most highly vascularized areas (hot spots) were selected in ×200 magnification fields. The mean of five counts was calculated and used in statistical analyses.

### Terminal deoxynucleotidyl transferase-mediated *deoxyuridine triphosphate* (dUTP)-biotin nick end labeling (TUNEL) assay

Apoptosis was measured in tissue sections by TUNEL assay. TUNEL assay allows easy demonstration of cell death as a result of apoptosis. The tissue samples were fixed in 4% paraformaldehyde for 24 h, dehydrated and embedded in paraffin in the conventional manner. The paraffin-embedded tissues were cut into 4 *μ*m-thick sections. Following deparaffinization in a graded alcohol series, the tissue sections were covered with 20 *μ*g/ml proteinase K PBS(−) for 15 min at room temperature, followed by blocking of endogenous peroxidase activity. The samples were then incubated with terminal deoxynucleotidyl transferase (TdT) enzyme and biotin-16-dUTP in TdT buffer containing 0.01% bovine serum albumin for 1.5 h at 37°C in a humidity chamber. Biotin-16-dUTP nucleotides that had been incorporated into DNA fragments were detected using the ABC method with diaminobenzidine (DAB) as the chromogen. In each tissue specimen, five high-power fields (magnification, ×400) were randomly selected. The apoptotic index was calculated in these fields as the percentage of positive cells, using the following equation: Apoptotic index = (number of positive cells/total number of cells) × 100% (23).

### Statistical analyses

Statistical analysis was performed with SPSS software version 17.0 (SPSS Inc., Chicago, IL, USA). Statistical significance among the control and drug-treated groups on tumor growth was determined by least significant difference (LSD) t-test. We used a Tukey’s honest significance difference (Tukey HSD) test for evaluation of the inhibitory activity on tumor cell MVD, VEGF mRNA expression and the increase in apoptosis. Correlations between VEGF score and MVD were estimated using the Karl Pearson coefficient of correlation. All experimental data were expressed as mean ± standard error (SE). P<0.05 was considered to indicate a statistically significant difference.

## Results

### Inhibition of ovarian cancer growth

**Figure 1:**
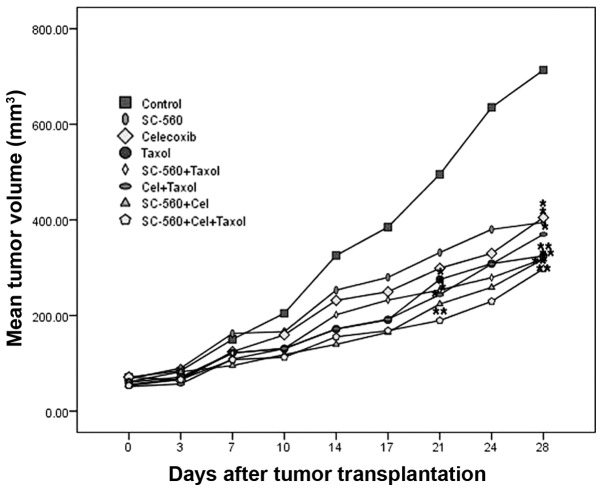
Effects of SC-560, celecoxib and Taxol on tumor growth *in vivo*. The effects of SC-560, celecoxib and Taxol on tumor growth were determined in an ovarian cancer model using SKOV-3 cells. After 7 days, to allow tumor establishment, mice were treated with SC-560, celecoxib and Taxol. Treatment was continued for 21 days. Average tumor size in all the drug-treated mice was significantly different from vehicle-treated mice at day 17, 21, 24 and 28. Statistical significance was determined using least significant difference (LSD) t-test. ^*^P<0.05; ^**^P<0.01, compared with the control.

To test whether SC-560, celecoxib or Taxol inhibits ovarian cancer growth, we used the human ovarian carcinoma cell line SKOV-3. The data in Fig. 1 show the relative effect of SC-560, celecoxib and Taxol treatment. The whole experiment was continued for 28 days. After 7 days to allow tumor establishment, mice were treated with SC-560, celecoxib and Taxol. The tumor growth increased in the control group whereas the growth was substantially suppressed in the treatment groups. After three weeks of treatment with SC-560, celecoxib or Taxol, a mean tumor volume of 331.72, 298.85 and 275.59 mm^3^ was observed, respectively, while the mean tumor volume of the control group was 495.30 mm^3^. At the end date of administration, all the treatment groups, with the exception of the SC-560 and celecoxib groups, had already demonstrated significant inhibitory effects on mean tumor volume (P<0.05). Moreover, at the end of the experiment, all treatment groups demonstrated notable effects on the inhibition of ovarian cancer growth; however, the inhibitory rate in these groups had no difference from each other (P>0.05).

### VEGF mRNA expression level

**Figure 2:**
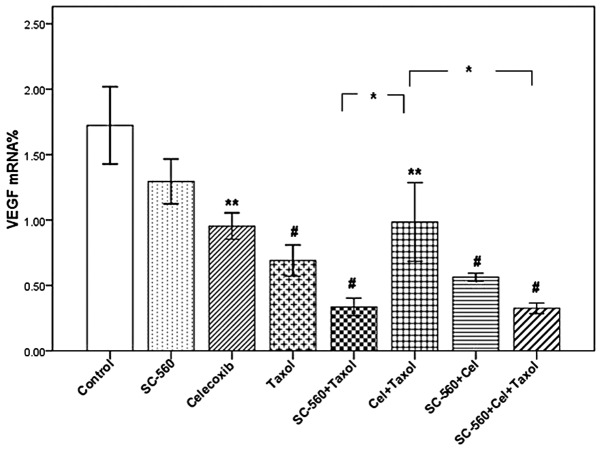
Effects of the drugs on the expression of VEGF mRNA. mRNA levels of VEGF were decreased in the treatment groups. ^*^P<0.05; ^**^P<0.01; ^#^P<0.001. Error bars indicate standard error. VEGF, vascular endothelial growth factor.

In this study, we measured VEGF mRNA levels in xenograft tumors by real-time PCR analysis. Three molecular isoforms of VEGF were generated by alternative splicing, rendering proteins containing 189, 165 and 121 amino acid residues. Real-time PCR analysis indicated the ΔCT of VEGF in the eight groups (Table I). As shown in Fig. 2, although the levels of VEGF mRNA in the SC-560/Taxol and SC-560/celecoxib/Taxol groups demonstrated a decreasing tendency when compared with the Taxol group, the difference was not statistically significant. However, the VEGF mRNA levels in these two groups were significantly lower than that in the celecoxib/Taxol group (P<0.05).

### Effect on tumor blood vessels

**Figure 3:**
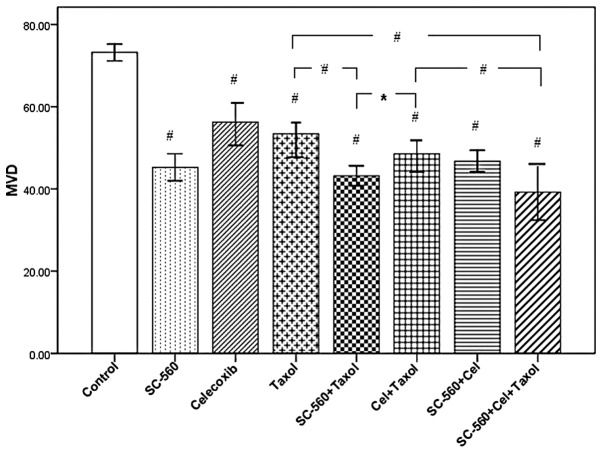
Effects of SC-560, celecoxib and Taxol on MVD *in vivo*. MVD of control and treatment groups illustrates the profound inhibitory effect of SC-560, celecoxib and Taxol on tumors. ^*^P<0.05; ^#^P<0.001. Error bars indicate standard error. MVD, microvessel density.

To evaluate the anti-angiogenic therapeutic efficacy of these three drugs, we histologically examined the residual tumors. Frozen tumor sections were immunohistochemically stained with an endothelial specific antibody against CD_34_. Immunohistochemical analysis identified a decrease in the number of CD_34_-positive microvessels of frozen tumor sections in mice treated with SC-560, celecoxib and Taxol. MVD in tumor tissues were reduced from 73.20±0.80 in the control group to 53.43±2.22, 43.20±0.94, 48.53±1.70 and 39.57±2.03 in the Taxol, SC-560/Taxol, celecoxib/Taxol and SC-560/celecoxib/Taxol-treated groups, respectively. The data in Fig. 3 show that sections from tumors in all drug-treated mice displayed a marked reduction in MVD compared with the vehicle-treated mice (P<0.001). In addition, the MVD values in the SC-560/Taxol and SC-560/celecoxib/Taxol groups displayed a distinguished reduction when compared with Taxol-treated mice (P<0.001), which were also significantly lower than that in the celecoxib/Taxol group (P<0.05 and P<0.001, respectively). However, there was no difference between the SC-560/Taxol and SC-560/celecoxib/Taxol groups.

### Correlation between VEGF and MVD

**Figure 4:**
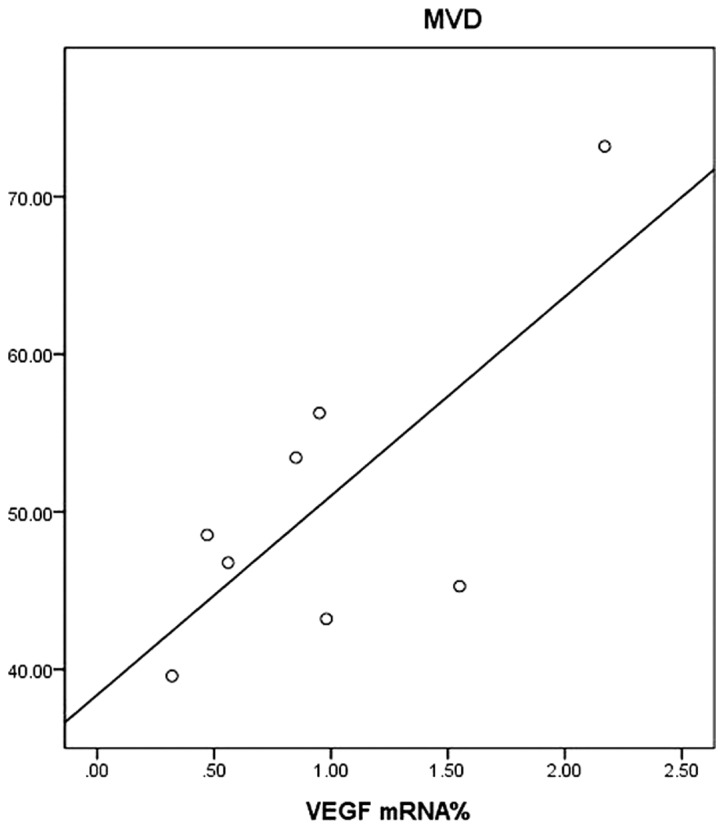
Correlation between the expressions of MVD and VEGF (r=0.737, P<0.05). MVD, microvessel density; VEGF, vascular endothelial growth factor.

Linear equations were created to show the correlation between MVD and VEGF (Fig. 4). The analysis revealed a positive correlation between the expressions of VEGF mRNA and MVD (correlation coefficient, r=0.737, P<0.05).

### Effect on tumor cell apoptosis

**Figure 5:**
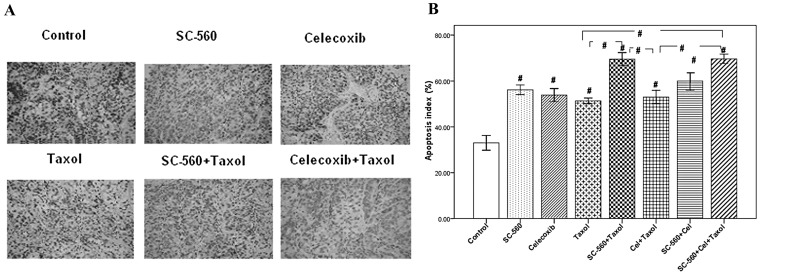
Cell apoptosis in xenograft tumors. (A) Immunostaining of cell apoptosis in tumors by TUNEL. The combination of COX selective inhibitor SC-560, celecoxib and Taxol accelerates tumor cell apoptosis. (B) The index of cell apoptosis was determined from the ratio of nuclear apoptosis-positive cells/total nuclei number. ^#^P<0.001. Error bars indicate standard error. TUNEL, terminal deoxynucleotidyl transferase-mediated deoxyuridine triphosphate nick end labeling; COX, cyclooxygenase.

We assessed cell apoptosis in the eight groups by TUNEL assay. Fig. 5A shows six representative images of tumor cell apoptosis. The number of apoptotic cells was more frequent in tumor sections of the treatment groups than in those of the control group. Data for the apoptotic index of the eight groups are shown in Fig. 5B. The apoptotic index in all drug-treated groups were significantly different to that of the control group (33.00±3.22%; P<0.001). The apoptotic indices in the SC-560/Taxol (69.50±2.87%) and SC-560/celecoxib/Taxol groups (69.67±2.08%) demonstrated a significant increase at the end of treatment compared with the Taxol group (51.33±1.26%; P<0.001). These were also significantly higher than that in the celecoxib/Taxol group (P<0.001). However, the apoptotic index between the SC-560/Taxol and SC-560/celecoxib/Taxol groups demonstrated no significant difference (P>0.05).

## Discussion

The main finding in the present study was that SC-560 enhances the anti-angiogenic and pro-apoptotic effect of Taxol and these effects are better than those observed with celecoxib.

In this study, the mean tumor volumes in the treatment groups were significantly lower than in the vehicle-treated mice at the end of treatment. The effects of SC-560 and celecoxib administered alone on inhibiting tumor growth were similar to that of Taxol. Taxanes are anti-microtubule agents that have strong anti-neoplastic effects. It is well known that Taxol is deemed to be the standard first-line therapy for patients with advanced ovarian cancer (5). A number of studies revealed that taxanes upregulate the COX-2 level in tumor cells and enhance MDR1 expression and functional activity (7,24); therefore, the addition of COX-2 inhibitors to Taxol is widely used for antitumor treatment (12,25,26). Sorokin identified that COX-2 inhibitors decrease the function of MDR1-enhanced accumulation of chemotherapy agents and decrease the resistance of tumors to chemotherapeutic drugs, thus enhancing the anti-tumor efficacy of Taxol (8). Furthermore, the combination of a COX-2-selective inhibitor and Taxol has been used in phase II trials of solid tumor treatment (27–29). However, research on Taxol in combination with COX-1-selective inhibitors used for the chemotherapy of ovarian cancer has not been conducted. COX inhibitors, which are selected based on definitive mechanisms relevant to tumorigenesis, have beneficial applications in human cancer chemoprevention trials. The combination of COX-1 and COX-2-selective inhibitors performed better anti-tumor effects than when administered alone (19,30). In this study, we added SC-560 and celecoxib to Taxol. Although the mean tumor volumes in the combination groups were significantly different from the vehicle-treated mice, no difference was observed between these groups and the Taxol group. This may be associated with the difference in dosage, the frequency of administration and the length of the experimental time. Therefore, this requires further investigation.

Ovarian cancer growth is angiogenesis-dependent and an increased production of angiogenic growth factors, including VEGF, is prognostically significant even during the early stages of the disease. VEGF is the most important of all the growth factors involved in tumor angiogenesis. Strong VEGF expression is suggested to play an important role in the tumor progression of ovarian carcinoma (31). A line of evidence reveals that levels of VEGF have been correlated with tumor response and survival rate in malignancies (32,33). In addition, the importance of angiogenesis in tumor progression has been highlighted, demonstrating that the angiogenic potential of tumors assessed by MVD directly correlates with poor prognosis (34). In the present study, we analyzed the levels of VEGF mRNA and values of MVD to assess the anti-angiogenic effect of these three drugs, as well as to observe whether combined administration produces better anti-angiogenic effects compared to a single administration. Our previous study identified that SC-560 inhibits the COX-associated upregulation of VEGF and reduces MVD (35). In the present study, the value of MVD in the SC-560/Taxol group was significantly different to the Taxol group and the level of VEGF mRNA in this group also demonstrated a decreasing tendency when compared with the Taxol group. In addition, the MVD value and VEGF mRNA level in the SC-560/Taxol group were significantly lower than that in the celecoxib/Taxol group. A number of studies identified that COX-1, not COX-2, mRNA and protein levels are elevated in human ovarian cancers. COX-1 is the dominant pathway responsible for generating PGs in epithelial ovarian cancers in mice. Additionally, COX-1 may contribute to carcinoma development in the ovary through stimulation of neovascularization and selective inhibition of COX-1, not COX-2, inhibits arachidonic acid-stimulated VEGF production (16–18,36). These results suggest that SC-560, when combined with Taxol, enhances the anti-angiogenic effect of Taxol and that this effect is better than with celecoxib treatment.

Unrestricted cell proliferation and reduced apoptosis are hallmarks of cancer cells (19). Apoptosis is a multistep process and an increasing number of genes have been identified to be involved in the control or execution of apoptosis (37). Taxanes induce an unbalance between microtubule polymerization and depolymerization, which finally leads to cell cycle arrest and apoptosis (38). COX inhibitors induce apoptosis by inhibiting the production of COXs, reducing the PGE_2_ levels and changing gene expression (39–42). In the present study, the apoptotic index in the SC-560/Taxol group was significantly different from the Taxol and celecoxib/Taxol groups, which suggests that SC-560 has a more pronounced effect on enhancing the proapoptotic activity of Taxol than celecoxib. This was consistent with the result that SC-560 had a greater effect on enhancing the anti-angiogenic effect of Taxol than celecoxib. This may be associated with the result observed by Gupta *et al* that COX-1, not COX-2, is overexpressed in ovarian cancer (16).

The present findings demonstrate that the combination of SC-560 and Taxol has a better effect on suppressing angiogenesis and promoting cell apoptosis than Taxol alone and these effects were better than the combination of celecoxib and Taxol.

## Figures and Tables

**Table I:** ΔCt of VEGF in the eight groups.

Group	VEGF 121	VEGF 165	VEGF 189
Control	6.94±0.23	4.58±0.26	6.34±0.23
SC-560	7.13±0.30	5.34±0.30	6.54±0.21
Celecoxib	7.10±0.23	5.19±0.20	7.99±0.11
Taxol	7.87±0.32	6.28±0.49	7.79±0.29
SC-560 + Taxol	9.14±0.33	7.00±0.48	8.99±0.23
Celecoxib + Taxol	8.31±0.64	4.60±0.43	8.04±0.40
SC-560 + celecoxib	8.50±0.22	5.91±0.21	8.05±0.25
SC-560 + celecoxib + Taxol	8.52±0.35	7.67±0.24	8.81±0.45

Molecular isoforms of VEGF are generated by alternative splicing, rendering proteins containing 189-, 165- and 121-amino acid residues. VEGF 189, 165 and 121 were routinely detected in this series of ovarian cancer. VEGF, vascular endothelial growth factor.

## References

[1] Ozols RF (2002). Future directions in the treatment of ovarian cancer. Semin Oncol.

[2] Yokoyama Y, Sakamoto T, Sato S, Saito Y (1999). Evaluation of cytoreductive surgery with pelvic and paraaortic lymphadenectomy and intermittent cisplatin-based combination chemotherapy for improvement of long-term survival in ovarian cancer. Eur J Gynaecol Oncol.

[3] Ozols RF (2002). Recurrent ovarian cancer: evidence-based treatment. J Clin Oncol.

[4] Kohler DR, Goldspiel BR (1994). Evaluation of new drugs: Paclitaxel (taxol). Pharmacotherapy.

[5] Pignata S, Scambia G, Ferrandina G (2011). Carboplatin plus paclitaxel versus carboplatin plus pegylated liposomal doxorubicin as first-line treatment for patients with ovarian cancer: the MITO-2 randomized phase III trial. J Clin Oncol.

[6] Wang Y, Qu Y, Niu XL, Sun WJ, Zhang XL, Li LZ (2011). Autocrine production of interleukin-8 confers cisplatin and paclitaxel resistance in ovarian cancer cells. Cytokine.

[7] Subbaramaiah K, Hart JC, Norton L, Dannenberg AJ (2000). Microtubule-interfering agents stimulate the transcription of cyclooxygenase-2. Evidence for involvement of ERK1/2 AND p38 mitogen-activated protein kinase pathways. J Biol Chem.

[8] Sorokin A (2004). Cyclooxygenase-2: potential role in regulation of drug efflux and multidrug resistance phenotype. Curr Pharm Des.

[9] Uddin S, Ahmed M, Hussain A, Assad L, Al-Dayel F, Bavi P, Al-Kuraya KS, Munkarah A (2010). Cyclooxygenase-2 inhibitior inhibits PI3-K/AKT kinase activity in epithelial ovarian cancer. Int J Cancer.

[10] Gu P, Su Y, Guo S, Teng L, Xu Y, Qi J, Gong H, Cai Y (2008). Over-expression of COX-2 induces human ovarian cancer cells (CAOV-3) viability, migration and proliferation in association with PI3-k/Akt activation. Cancer Invest.

[11] Athanassiadou P, Grapsa D, Athanassiades P, Gonidi M, Athanassiadou AM, Tsipis A, Patsouris E (2008). The prognostic significance of COX-2 and survivin expression in ovarian cancer. Pathol Res Pract.

[12] Ferrandina G, Ranelletti FO, Martinelli E, Paglia A, Zannoni GF, Scambia G (2006). Cyclooxygenase-2 (Cox-2) expression and resistance to platinum versus platinum/paclitaxel containing chemotherapy in advanced ovarian cancer. BMC Cancer.

[13] Masferrer JL, Leahy KM, Koki AT, Zweifel BS, Settle SL, Woerner BM, Edwards DA, Flickinger AG, Moore RJ, Seibert K (2000). Antiangiogenic and antitumor activities of cyclooxygenase-2 inhibitors. Cancer Res.

[14] Olsen SR (2005). Taxanes and COX-2 inhibitors from molecular pathways to clinical practice. Biomed Pharmacother.

[15] Merchan JR, Jayaram DR, Supko JG, He X, Bubley GJ, Sukhatme VP (2005). Increased endothelial uptake of paclitaxel as a potential mechanism for its antiangiogenic effects: potentiation by COX-2 inhibition. Int J Cancer.

[16] Gupta RA, Tejada LV, Tong BJ, Das SK, Morrow JD, Dey SK, Dubois RN (2003). Cyclooxygenase-1 is overexpressed and promotes angiogenic growth factor production in ovarian cancer. Cancer Res.

[17] Daikoku T, Wang D, Tranguch S, Morrow JD, Orsulic S, DuBois RN, Dey SK (2005). Cyclooxygenase-1 is a potential target for prevention and treatment of ovarian epithelial cancer. Cancer Res.

[18] Daikoku T, Tranquch S, Trofimova IN, Dinulescu DM, Jacks T, Nikitin AY, Connolly DC, Dey SK (2006). Cyclooxygenase-1 is overexpressed in multiple genetically engineered mouse models of epithelial ovarian cancer. Cancer Res.

[19] Li W, Wang J, Jiang HR, Xu XL, Zhang J, Liu ML, Zhai LY (2011). Combined effects of cyclooxygenase-1 and cyclooxygenase-2 selective inhibitors on ovarian carcinoma in vivo. Int J Mol Sci.

[20] Williams CS, Watson AJ, Sheng H, Helou R, Shao J, DuBois RN (2000). Celecoxib prevents tumor growth in vivo without toxicity to normal gut: lack of correlation between in vitro and in vivo models. Cancer Res.

[21] Gerdes J, Lemke H, Baisch H, Wacker HH, Schwab U, Stein H (1984). Cell cycle analysis of a cell proliferation associated human nuclear antigen defined by the monoclonal antibody Ki-67. J Immunol.

[22] Weidner N, Folkman J, Pozza F, Bevilacqua P, Allred EN, Moore DH, Meli S, Gasparini G (1992). Tumor angiogenesis: a new significant and independent prognostic indicator in early-stage breast carcinoma. J Natl Cancer Inst.

[23] del Vecchio MT, Leoncini L, Buerki K, Kraft R, Megha T, Barbini P, Tosi P, Cottier H (1991). Diffuse controcytic and/or centroblastic malignant non-Hodgkins lymphomas: comparison of mitotic and pyknotic (apoptotic) indices. Int J Cancer.

[24] Ratnasinghe D, Daschner PJ, Anver MR, Kasprzak BH, Taylor PR, Yeh GC, Tangrea JA (2001). Cyclooxygenase-2, P-glycoprotein-170 and drug resistance; is chemoprevention against multidrug resistance possible?. Anticancer Res.

[25] Altorki NK, Keresztes RS, Port JL, Libby DM, Korst RJ, Flieder DB, Ferrara CA, Yankelevitz DF, Subbaramaiah K, Pasmantier MW, Dannenberg AJ (2003). Celecoxib, a selective cyclo-Oxygenase-2 inhibitor, enhances the response to preoperative paclitaxel and carboplatin in early-stage non-small-cell lung cancer. J Clin Oncol.

[26] Gasparini G, Meo S, Comella G, Stani SC, Mariani L, Gamucci T, Avallone A, LoVullo S, Mansueto G, Bonginelli P, Gattuso D, Gion M (2005). The combination of the selective cyclooxygenase-2 inhibitor celecoxib with weekly paclitaxel is a safe and active second-line therapy for non-small cell lung cancer a phase II study with biological correlates. Cancer J.

[27] Altorki NK, Christos P, Port JL, Lee PC, Mirza F, Spinelli C, Keresztes RS, Beneck D, Paul S, Stiles BM, Zhang Y, Schrump DS (2011). Preoperative taxane-based chemotherapy and celecoxib for carcinoma of the esophagus and gastroesophageal junction: results of a phase 2 trial. J Thorac Oncol.

[28] Bhatt RS, Merchan J, Parker R, Wu HK, Zhang L, Seery V, Heymach JV, Atkins MB, McDermott D, Sukhatme VP (2010). A phase 2 pilot trial of low-dose, continuous infusion, or “metronomic” paclitaxel and oral celecoxib in patients with metastatic melanoma. Cancer.

[29] Mutter R, Lu B, Carbone DP, Csiki I, Moretti L, Johnson DH, Morrow JD, Sandler AB, Shyr Y, Ye F, Choy H (2009). A phase II study of celecoxib in combination with paclitaxel, carboplatin, and radiotherapy for patients with inoperable stage IIIA/B non-small cell lung cancer. Clin Cancer Res.

[30] Kitamura T, Itoh M, Noda T, Matsuura M, Wakabayashi K (2004). Combined effects of cyclooxygenase-1 and cyclooxygenase-2 selective inhibitors on intestinal tumorigenesis in adenomatous polyposis coli gene knockout mice. Int J Cancer.

[31] Yamamoto S, Konishi I, Mandai M, Kuroda H, Komatsu T, Nanbu K, Sakahara H, Mori T (1997). Expression of vascular endothelial growth factor (VEGF) in epithelia ovarian neoplasms: correlation with clinicopathology and patient survival and analysis of serum VEGF levels. Br J Cancer.

[32] Carmeliet P, Jain RK (2000). Angiogenesis in cancer and other diseases. Nature.

[33] Folkman J (2007). Angiogenesis: an organizing principle for drug discovery?. Nat Rev Drug Discov.

[34] Weidner N, Semple JP, Welch WR, Folkman J (1991). Tumor angiogenesis and metastasis - correlation in invasive breast carcinoma. N Engl J Med.

[35] Li W, Xu RJ, Lin ZY, Zhuo GC, Zhang HH (2009). Effects of a cyclooxygenase-1-selective inhibitor in a mouse model of ovarian cancer, administered alone or in combination with ibuprofen, a nonselective cyclooxygenase inhibitor. Med Oncol.

[36] Dore M, Cote LC, Mitchell A, Sirois J (1998). Expression of prostaglandin G/H synthase type 1, but not type 2, in human ovarian adenocarcinomas. J Histochem Cytochem.

[37] Gastman BR (2001). Apoptosis and its clinical impact. Head Neck.

[38] Foa R, Norton L, Seidman AD (1994). Taxol (paclitaxel): A novel antimicrotubule agent with remarkable anti-neoplastic activity. Int J Clin Lab Res.

[39] Leahy KM, Ornberg RL, Wang Y, Zweifel BS, Koki AT, Masferrer JL (2002). Cyclooxygenase-2 inhibition by celecoxib reduces proliferation and induces apoptosis in angiogenic endothelial cells in vivo. Cancer Res.

[40] Daikoku T, Tranguch S, Chakrabarty A, Wang D, Khabele D, Orsulic S, Morrow JD, Dubois RN, Dey SK (2007). Extracellular signal-regulated kinase is a target of cyclooxygenase-1-peroxisome proliferator-activated receptor-delta signaling in epithelial ovarian cancer. Cancer Res.

[41] Bottone FG, Martinez JM, Alston-Mills B, Eling TE (2004). Gene modulation by Cox-1 and Cox-2 specific inhibitors in human colorectal carcinoma cancer cells. Carcinogenesis.

[42] Zhu J, Song X, Lin HP, Young DC, Yan S, Marquez VE, Chen CS (2002). Using cyclooxygenase-2 inhibitors as molecular platforms to develop a new class of apoptosis-inducing agents. J Natl Cancer Inst.

